# Multiple Amino Acid Sequence Alignment Nitrogenase Component 1: Insights into Phylogenetics and Structure-Function Relationships

**DOI:** 10.1371/journal.pone.0072751

**Published:** 2013-09-03

**Authors:** James B. Howard, Katerina J. Kechris, Douglas C. Rees, Alexander N. Glazer

**Affiliations:** 1 Department of Biochemistry, Molecular Biology, and Biophysics, University of Minnesota, Minneapolis, Minnesota, United States of America; 2 Division of Chemistry and Chemical Engineering, California Institute of Technology, Pasadena, California, United States of America; 3 Department of Biostatistics and Informatics, Colorado School of Public Health, Aurora, Colorado, United States of America; 4 Division of Chemistry and Chemical Engineering, Howard Hughes Medical Institute, California Institute of Technology, Pasadena, California, United States of America; 5 Department of Molecular and Cell Biology, University of California, Berkeley, California, United States of America; University of South Florida College of Medicine, United States of America

## Abstract

Amino acid residues critical for a protein's structure-function are retained by natural selection and these residues are identified by the level of variance in co-aligned homologous protein sequences. The relevant residues in the nitrogen fixation Component 1 α- and β-subunits were identified by the alignment of 95 protein sequences. Proteins were included from species encompassing multiple microbial phyla and diverse ecological niches as well as the nitrogen fixation genotypes, *anf*, *nif*, and *vnf*, which encode proteins associated with cofactors differing at one metal site. After adjusting for differences in sequence length, insertions, and deletions, the remaining >85% of the sequence co-aligned the subunits from the three genotypes. Six Groups, designated Anf, Vnf , and Nif I-IV, were assigned based upon genetic origin, sequence adjustments, and conserved residues. Both subunits subdivided into the same groups. Invariant and single variant residues were identified and were defined as “core” for nitrogenase function. Three species in Group Nif-III, *Candidatus Desulforudis audaxviator*, *Desulfotomaculum kuznetsovii*, and *Thermodesulfatator indicus*, were found to have a seleno-cysteine that replaces one cysteinyl ligand of the 8Fe:7S, P-cluster. Subsets of invariant residues, limited to individual groups, were identified; these unique residues help identify the gene of origin (*anf*, *nif*, or *vnf*) yet should not be considered diagnostic of the metal content of associated cofactors. Fourteen of the 19 residues that compose the cofactor pocket are invariant or single variant; the other five residues are highly variable but do not correlate with the putative metal content of the cofactor. The variable residues are clustered on one side of the cofactor, away from other functional centers in the three dimensional structure. Many of the invariant and single variant residues were not previously recognized as potentially critical and their identification provides the bases for new analyses of the three-dimensional structure and for mutagenesis studies.

## Introduction

In their pioneering paper, “Molecules as Documents of Evolutionary History”, Zuckerkandl and Pauling [1] reasoned that comparison of homologous polypeptide chains provided ways of gaining information about their evolutionary history, and the value of “the study of three-dimensional models, to permit one to make predictions about the effect of particular substitutions.” They substantiated these insights by examining the small number of available hemoglobin sequences and the low resolution hemoglobin crystal structure [2]. Fitch and Margoliash [3], in their seminal study, developed the phylogenetic feature of multiple sequence alignment to construct a tree comparing cytochrome C from diverse species, encompassing more than a billion years of evolution. A second important application of multiple sequence alignment is to identify highly conserved residues in a protein family and to evaluate these residues in high resolution crystal structures with respect to their importance in the protein structure and function. The proteins of nitrogen fixation are excellent candidates for study by this approach: there are many known and putative nitrogen fixing species represented across the full spectrum of microbial diversity; there is a large, whole genome database for potential sequences; and there are multiple high-resolution crystal structures for the proteins.

Nitrogen fixation – reduction of dinitrogen to ammonia–is the primary path for replenishment of ammonia in the nitrogen cycle, yet this capability is limited to bacteria and Archaea. While the genes for the nitrogen fixation enzymes are widely distributed, they are not universally found and are a well-documented example of horizontal gene transfer between phylogenetically well-separated organisms [4]. Nitrogenase is composed of two proteins, commonly referred as Component 1 and Component 2. Component 2 (Fe-protein) binds and hydrolyzes two ATP while transferring electrons to Component 1, which contains the active site for dinitrogen reduction. Because multiple electrons are required for dinitrogen reduction, the two protein components undergo multiple cycles of association and dissociation for the inter-protein electron transfer steps [5].

**Figure 1:**
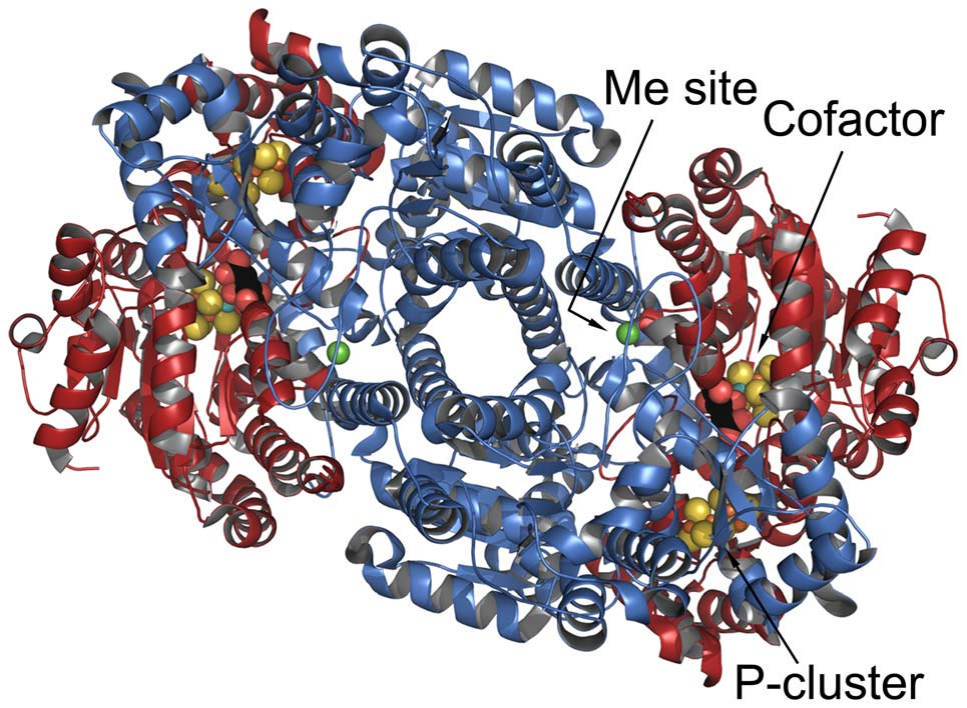
Three-dimensional structure of the α_2_β_2_ tetramer of *A. vinelandii* Component 1 (3U7Q.pdb). The figure is centered on the approximate two-fold axis between the αβ pairs. Red is the α-subunit and blue is the β-subunit with the three metal centers shown in space filling PCK models. The Component 2 (Fe-protein) docking site is along the axis (arrow) identifying the P-cluster. Figure was prepared using Pymol (http://pymol.org/).

The three dimensional structures of Components 1 and 2 as well as of several complexes between the two components have been determined for the proteins from three species including that for the *Azotobacter vinelandii* Component 1 at 1.0 Å [6]–[13]. Component 1 is an α_2_β_2_ tetramer of two related but different subunits where the two β subunits, β–β′, form a two-fold symmetry core with an α-subunit uniquely associated with each β-subunit, as shown in Figure 1 [6], [7], [10]. Component 1 has two unique Fe:S based clusters, the 8Fe:7S P-cluster and the 7Fe:M: 9S:C:homocitrate cofactor where M can be Mo, V or another Fe atom. The P-cluster is shared at the interface of the α-β pair and can be considered two 4Fe:4S clusters fused at a common corner S with two bridging and four terminal cysteinyl ligands [14]. The cofactor, fully embedded with one in each α-subunit, is more complex having eight metals resembling the fusion of two clusters bridged by inorganic sulfides. At one corner the alternate Mo, V, or Fe atom is coordinated by a histidyl residue and the organic acid, homocitric acid. Central to the cofactor structure is an interstitial carbon atom hexacoordinated to six equidistant Fe atoms [6], [10]. Because this ensemble of the cluster and homocitric acid can be extracted intact from denatured protein, it has been called a cofactor and is abbreviated, Fe(Mo, V, or Fe) co [15]. This arrangement suggests that each α-β pair is an independent electron transfer and substrate-reducing unit. The present understanding of the reaction sequence is that electrons are transferred from the Fe-protein 4Fe:4S cluster to the P-cluster and finally to the cofactor for substrate reduction [5] (see Figure 1 for relative positions of metal centers and Component 2 binding site).

The earliest forms of Component 1 were isolated from *A. vinelandii*, *Klebsiella pneumoniae*, and *Clostridium pasteurianum* and were found to contain Mo [16]. Subsequently, the genes for the three structural peptide chains that constitute Components 1 and 2 were identified as *nifH* (the two identical subunits of Component 2), *nifD* (Component 1 α-subunit), and nif*K* (Component 1 β-subunit) (reviewed in [17]). In the *A. vinelandii* nitrogenase gene cluster, two other copies of homologous structural genes were found and based upon selected growth conditions, each of the structural genes was expressed [18]–[24]. These alternative nitrogenases were distinguished as containing cofactors with either V or only Fe but not Mo [25]. Although the three forms are encoded as genetically distinct structural proteins, Nif (Mo containing), Vnf (V containing), and Anf (Fe only) proteins, they are, nevertheless, highly similar proteins and are considered part of a common family [26]. Indeed, each cofactor type can be extracted and inserted into any of the three distinct cofactor-deficient parent proteins resulting in active Component 1 [25]. All nitrogen fixing species appear to have the *nif* system while less than one fourth of the species identified to date contain the additional, alternate *vnf* and/or *anf* genes.

A number of studies have emphasized indices of similarity between paralogs and orthologs in the broad nitrogenase family to define several different subclasses and to suggest paths for the natural history, microbial distribution, and evolution of the system. For example, Fani et al. [27] and Raymond et al. [28] defined, in addition to three classes of *nifD/K*, multiple groups of paralogous genes including those for cofactor biosynthesis (*nif E/N*) and for bacteriochlorophyll and chlorophyll biosynthesis. Boyd et al. [29] extended these studies to propose an alternate path for evolution of the groups within the family. In our study, the focus is on the evaluation of individual amino acids in the structure-function of Component 1, and to this end, we have assembled a multiple protein sequence alignment limited to the three genotypes encoding Component 1. Following the precepts of Zuckerkandl and Pauling [2] that natural selection retains essential residues, we have cataloged the Component 1 residues and have identified the most conserved residues, namely, the invariant and single variant residues. These residues define a common “core” of nitrogenase Component 1 that can be evaluated, ultimately using the three-dimensional protein structure, in exploration of a common structure-function. Furthermore, the constraints of invariance allow significant new insights to phylogenetic analyses.

## Methods

Amino acid sequences for nitrogenase structural proteins were obtained from the NCBI DNA data repository (www.ncbi.nlm.gov). Taxonomic assignments were obtained from the NCBI Taxonomy Browser (www.ncbi.nlm.nih.gov/Taxonomy/Browser/wwwtax.cgi). The initial data set built on that reported by Glazer and Kechris [30] and was expanded by Basic Local Alignment Search Tool (BLAST^®^) using the protein probes NifD, AnfD, or VnfD from *A. vinelandii* and NifD from *C. pasteurianum* (see Table S1 for accession numbers). As Groups III and IV (see below) were defined, search for additional members of these groups used the NifD of a local group member. The data set was evaluated in several steps to insure broad distribution of microbial species. Sequences were taken from whole genomes with older sequences updated as genomes became available. Generally, to reduce bias in the data, only one member of a genus was chosen. The data set was expanded to include the K gene (encoding the β-subunit) for each of the corresponding D genes (we use the terms D and K gene to be inclusive of *nif, anf and vnf* families).

We note several potential sources for errors in our data set that can arise from using translation of the large DNA database for aligning the nitrogenase proteins:

1. The DNA sequences are subject to technical errors of the sequencing process including colony selection for DNA extraction and amplification.
2. The colony selected has not been rigorously demonstrated to have the enzymatic activity attributed to the gene. That is, the DNA may harbor mutations not representative of the wild-type species.
3. Gene annotations and identification are varied, confusing, and occasionally incorrect in the gene database (see example discussed below). Thus, diligence is required to cross check the identity of each gene added to the analysis.
4. Species strain identification and naming is subject to change.

The protein sequences were analyzed with ClustalX_v2.0 [31] using the default parameters; the output was as graphic and as text alignment. The latter was imported to a MS Excel^®^ spreadsheet and the sequences were numbered to correspond to the *A. vinelandii* proteins in the crystal structures. This numbering is used throughout the analysis. In the spreadsheet, to compensate for extensions, insertions, and deletions compared to the *A. vinelandii* sequence, deletions are blank cells in the other sequences and insertions are blank cells retaining the same residue number in *A. vinelandii* until the register is re-established. The positions of insertions, deletions, and extensions were consistent with loops in the three-dimensional structure and would be unlikely to disrupt the larger protein fold. As new sequences were added, the entire data set was realigned as a unit with final spreadsheets containing 95 sequences from 75 different species for the α-subunit (NifD, AnfD, VnfD) and for the β-subunit (NifK, AnfK, VnfK).

16S rRNA sequences for the species were obtained by searching the NCBI Gene database using “16S rRNA” as the search term. For ten of the entries, this search did not provide a sequence and the same search was performed using the NCBI Nucleotide database. In many of the searches, at least 2 possible entries were returned, which were often the same sequence. When different sequences were returned, the most frequent sequence was selected. In three cases, when the exact strain was not available, an alternative strain for the same species was used. Phylogenetic trees were constructed in Phylip 3.69 using default options (http://evolution.genetics.washington.edu/phylip.html). One hundred bootstrap samples were created using the “seqboot” function. Distances between the 16S rRNA sequences were calculated using “dnadist” and were used to build neighbor joining trees with the “neighbor” function for each bootstrap sample. A consensus tree was determined with the “consense” function and trees were displayed using “drawtree” at http://mobyle.pasteur.fr/cgi-bin/portal.py. The tree file was imported into Microsoft Powerpoint to add text and additional labels.

Calculations of inter-atomic distances for amino acid residues used the 1.16 Å coordinates (file 1M1N.pdb) and CCP4 [32].

## Results and Discussion

At the outset, it should be stated that invariant or low variant sites as signatures in multi-sequence alignment are open to revision as new sequences are added. As our study progressed and new sequences were added to expand the phylogenic and ecological range of the included organisms, it was pleasantly surprising that the patterns described below changed only marginally. The main changes observed were that a few residues moved from invariant to single variant class. Indeed, there were no changes to these two classes or the “strong motifs” (see discussion below) when the last eight sequences were added to expand the range of divergent sources.

**Figure 2:**
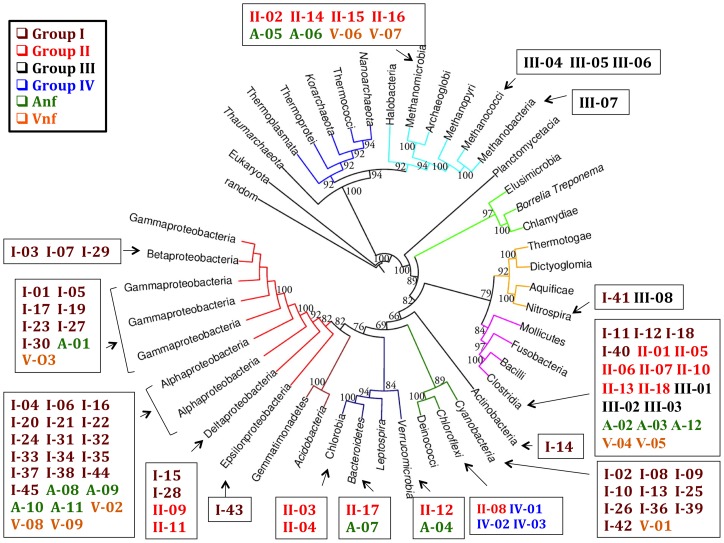
Phylogeny of species used for multi-sequence alignment of NifD and NifK. The species in the data analysis set (identifiers and species are in Table S1) were superimposed on a simplified whole-proteome tree from Jun et al. (Figure 2 in [34], constructed with whole proteomes of 884 prokaryotes). Identifiers are based upon the six nitrogenase groups; species with both Nif and either Anf or Vnf have more than one identifier.

For critical residues to be revealed by natural selection, a fundamental requirement is that the species used in the multiple sequence alignment represent a broad, distinctive phylogenetic distribution. Although the number of known species with putative nitrogen fixation genes greatly exceeds the 75 species used here (e.g., [33]), the criteria for inclusion of the species were that whole genomes are available, that a broad range of classes is represented, and that the species exemplify metabolic diversity and distinctive ecological niches. One goal of this study is to correlate the sequences of the three known genetic variants of nitrogenase which also have different apparent metal requirements in the cofactor. When Anf and Vnf versions of Component 1 were available, the Nif sequences from the same species were included. The diversity of species in our analysis is indicated by the distribution of these species across nearly the whole proteome map of Jun et al. [34] as shown in Figure 2. Their tree was constructed based on analyzing 884 full genomes and independent of the ability of a species to fix nitrogen. For our purpose, we have superimposed the species from our study on a simplified version of their map to show the distribution in the larger microbial world. A second demonstration of the species distribution is shown in Figure S1 constructed independently using the 16S rRNA similarity index for just the species in our data set. Jun et al. [34] observed that, with some important exceptions, there is good agreement between these two types of maps of the microbial world. However, we found some potentially interesting differences when the nitrogen fixation genes are considered. These differences may reflect the lower resolution of the 16S rRNA map as well as horizontal gene transfer [4].

The alignments of the proteins encoded by D and K genes immediately verified that Nif, Anf, and Vnf proteins are homologous and fully align with a consensus α-subunit and a consensus β-subunit. Although, as we show below, the three protein families can be distinguished and identified by separate conserved amino acid groups, the larger pattern is for a single protein family that likely has a common core or fundamental three-dimensional structure. Deviations from the core structure, suggested by the primary sequence variance and insertion/deletions, are to be expected while the core structure is maintained. The three dimensional structures of Component 1 from *A. vinelandii* and *C. pasteurianum* exemplify how the core is maintained despite several insertions/deletions including a 52 residue insertion in the *C. pasteurianum* protein; the two proteins have similar protein fold patterns with a large superimposed structural core (RMS 1.6 Å) [8]. Hence, we consider it justified to initially treat the sequences from the three gene families as one.

### Identification of invariant, single variant and, double variant residues

**Figure 3:**
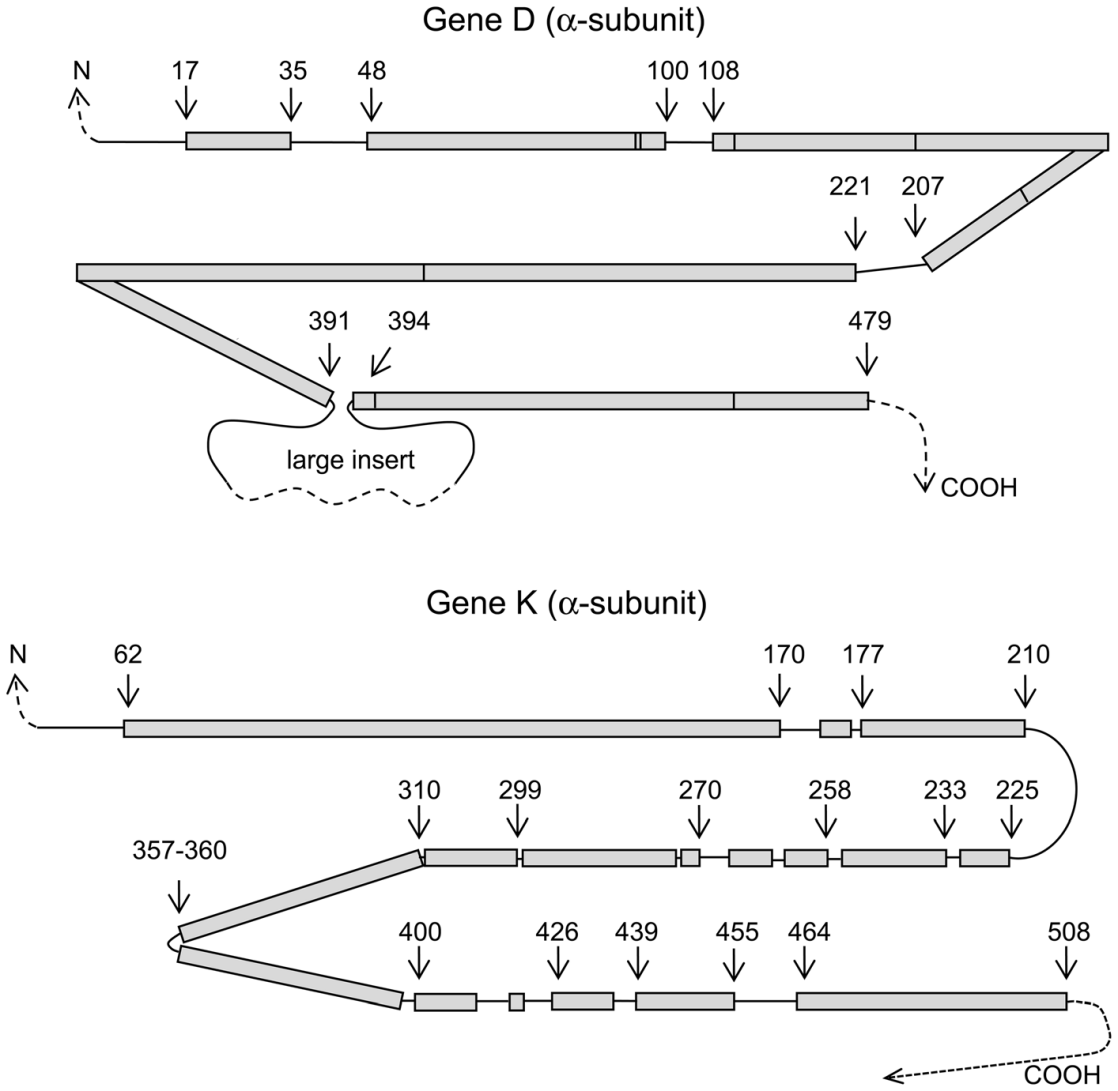
Diagram showing co-aligned regions of gene D and gene K used to identify amino acid variants. Shaded blocks are the regions co-aligned across all 95 sequences. Lines between blocks have one or more insertions or deletions and are not included in the co-alignment. Numbering is based upon the *A. vinelandii* proteins. Gene D and Gene K co-aligned residues are explicitly given in Table S2.

Numerous algorithms have been devised to identify putative functional elements or motifs using a statistical analysis of multiple sequence alignment, often coupled to energy minimization calculations (for example, [35]–[39]). Use of the spreadsheet alignment based on ClustalX v2.0 requires minimal manipulation of the data that can be easily expanded with new sequences and searched by simple spreadsheet counting functions. Both the α- and β-subunits have substantial variation in length, as shown in Figure 3, that includes extensions at the terminals as well as insertions and deletions. The extensions, insertions and deletions likely have important but more limited roles characteristic of subgroups, for example Anf and Vnf families appear to have a third, low molecular weight component for stabilization of the tetrameric organization [25], [40]. Hence, the fully co-linear regions more generally define the central structure-function elements of nitrogenase. For the most part, the chain length variations are clustered in sets of sequences and, as discussed below, help to identify the classes or Groups of nitrogenase. Excluding variations in size, there are 422 residues in the α-subunit and 386 residues in the β-subunit that align across all 95 sequences (Table 1). Within the common sequence alignment (shown as blocks in Figure 3 with an explicit list of the co-aligned residue numbers used in our analysis given in Table S2), a nucleus of invariant and single variant residues accounts for only ∼17% of the common co-aligned structure (808 residues for the combined the α- and β-subunits). In contrast, >65% of the co-aligned sequence positions have five or more different amino acids including >45% highly variable positions with 7–15 different amino acids. The high variance rate for much of the sequence is strong evidence that each sequence position has been subjected to genetic modification and that natural selection has retained a critical core of residues as invariant or single variants. Furthermore, the invariant residues are encoded by their available codons, for example, invariant α-Arg60 is encoded by at least five of the six arginine codons, which suggests that natural selection has preserved the core residues even as species specific codon utilization was imposed.

**Table 1:** Invariant and Single Variant Residues.

	α-subunit	β-subunit
Sequence size^1^	462–578	454–548
Aligned residues^2^	422	386
Invariant residues	41	27
% invariant^3^	9.7%	7.0%
Total Single variant	39	33
% single variant^3^	9.2%	8.5%

Values are for 95 aligned Nif, Anf, and Vnf sequences.

1 Range of full sequence lengths.

2 Residues common to *nif, anf, vnf* exclusive of extensions, insertions or deletions.

3 Based upon total number of aligned residues.

In addition to the invariant residues, the single variant residues are considered critical to the structure-function core. These residues with sequence positions are given in Tables S3 and S4. Three types of single variant positions can be identified: a) a single amino acid is found in 94 of 95 sequences; b) two functionally similar amino acids are found; and c) two, apparently, functionally dissimilar amino acids are found. In the first case, some outlier residues could be potential sequencing errors in that the amino acid occurred only once in the 95 sequences, was encoded by a codon that differed by a single base from one of the dominant amino acid codons, and was functionally different, e.g., α-Asp161, α-His196, α-Phe316, α-Gly348, and α-Gly455. Other single outlier variants are more difficult to assign as errors because both amino acids were functionally similar or the codons for the two residues were not single base differences. Despite these potential reservations, all residues used in our analysis were as given in the translated gene data base.

In addition to the core invariant and single variant residues, double variant sites (three different amino acids at a sequence position), and a few notable examples where there are a high number of substitutions (4–6) yet one amino acid dominates >90% (>85/95 sequences) are included in the tables for completeness. Our restricted assignment of critical core residues does not exclude possibly important sites that have higher variance but where the substitutions are generally functionally equivalent, nor are we evaluating possible compensating, suppressor substitutions. Indeed, although single variant residues are deemed critical to the enzyme structure-function, even these residues may have been rescued by covariance at another site (see example below). In contrast, by definition, invariant residues have not been rescued by covariance at suppressor sites; the criterion of natural selection suggests that invariant residues have been tested and a change elsewhere cannot provide the required compensating property of the invariant residue.

There are several general patterns evident in the amino acid alignment across all 95 sequences of *nif*, *anf* and *vnf* origin:

a. The α- and β-subunits are paralogues with strong similarity in three dimensional fold and share the P-cluster and Component 2 (Fe-protein) binding site (see Figure 1) [7], [13]. However, the α-subunit contains a larger number of core residues compared to the β-subunit which likely reflects the higher structural restraint imposed by the cofactor interactions and associated electron transfer pathways. As seen in Figure 3, the α-subunit has half the number of insertion/deletion interruptions in the sequence compared to the β-subunit, although the α-subunit has the largest continuous insertion in some sequences.

b. As shown in Tables S3 and S4, the use and distribution of amino acid types are asymmetric in the core of the two paralogous subunits. Although the aliphatic amino acids leucine, isoleucine and valine were invariant in some sites, there are no examples in either subunit of an invariant methionine, tryptophan, alanine, or threonine which also have hydrophobic properties and unique structural characteristics. Glycine is dominant in both the α- and β-subunit invariant-single variant classes making up 35% of invariant residues and 21% of dominant single variants. The large number of glycine residues is likely a consequence of its unique functional roles in peptide chain turns, close packing between chains, close packing around ligands at metal centers, and cis peptide conformation. All four of these properties are exhibited in the structure. Invariant arginine predominates over lysine by 7 to 1 in the two subunits; likewise aspartic acid predominates over glutamic acid 6 to 2. There are four invariant histidine in the α-subunit yet there are none in the β-subunit. Noticeable is the paucity of invariant aromatic residues, no tryptophan, three phenylalanine, and only one tyrosine between the two subunits.

c. There are several examples of amino acid residues that are invariant in one position while paired as a single variant with an iso-structural amino acid in other positions. Two leucine, two isoleucine, and two valine in the two subunits were invariant yet, in the case of isoleucine and valine, they were paired five times as single variants, while never paired with leucine (Tables S3 and S4). Two examples serve to emphasize the stringent requirements for otherwise similar residues. α-Leu158 and α-Ile159 are neighbors and are invariant while α-Val/Ile123 and α-Val/Ile124 are likewise neighbors but are single variants with all four sequence combinations. This strongly argues that in some sequence specific sites there is a highly precise structural requirement, while in other sites either of the β-branched aliphatic amino acids is acceptable. A second intriguing example is the arginine and lysine pair; both amino acids are invariant in some sites while they can substitute for each other at other locations. At position α-96, 72 of the 95 sequences have arginine (23/95 sequences as lysine). Inspection of the crystal structure shows the α-Arg96 side chain is in the cofactor inter shell and has three H-bonds, two to the peptide backbone of α-Gly69-α-Val70 and one to the side chain α-Asn98. α-Asn98 is a five variant residue, yet when α-96 is lysine, α-98 is uniquely tyrosine. Whether tyrosine is a compensating rescue for the lysine substitution would be conjecture, it does provide a potential H-bond to the α-Gly69-α-Val70 backbone. This covariant pair, α-Lys96/α-Tyr98, is universal in Anf and Vnf sequences but is also found in some Nif Group III sequences (see below for Group designations) and may reflect the evolutionary differences between groups described below.

### Nitrogenase groups

Three types or groups of nitrogenase are evident from the genetics as encoded by *nif*, *anf*, and *vnf*. Although the alignment indicates a strong homology at the core residues, the three protein families, Nif, Anf, and Vnf are treated at the next level as separate Groups. In addition, the Nif family has long been recognized to have two subgroups exemplified by *A. vinelandii* and *C. pasteurianum* Component 1 where the α-subunit has a large 52 residue insertion at residue 391 of the *A. vinelandii* sequence (see Figure 3, Table S2) [8], [41]. The insertion as an independent loop is verified by the crystal structures of the two proteins where the loop is on one surface of the α-subunit [8]. In our data set, 18 sequences were identified as having this insertion and were classified as Group II. The remaining *nif* nitrogenase protein sequences, those without the large α-subunit insertion, can be further divided into Groups I, III, and IV by several criteria. Group I, the largest group in number, resembles *A. vinelandii* sequences. Group I members also are identified by a longer amino terminal of the β-subunit (measuring from the first cysteinyl ligand of the P-cluster, β-Cys70 in *A. vinelandii*); the extended β-subunit contacts and covers a segment of the α-subunit which is exposed in the *C. pasteurianum* α-subunit [8]. The Groups I, III, IV were further distinguished by other smaller insertions and deletions in both the α- and β-subunits and these patterns of chain differences were preserved when representative group specific sequences were used in additional BLAST searches, namely, Group I based upon *A. vinelandii*, Group III based upon *Methanococcus aeolicus*, and Group IV based upon *Roseiflexus castenholzii*. It should be emphasized that the α- and β-subunits independently subdivided into the same groups suggesting the two subunits have followed a similar evolutionary history. This strengthens the justification for the subdivisions. In our species selection, the six groups are not equally populated (See Table S1 for species in each group); Group I is conspicuously the largest (45/95 sequences) although Group II is well represented with 18 examples. Group III could have been expanded to at least 12 by including several sequences from the same genus. For example, genomes are reported for eight *Caldicellulosiruptor* species which are tightly grouped by 16S-rRNA analysis [42] . Four of the species have *nif* genes with virtually identical NifD/K sequences and we have included only III-01, *Caldicellulosiruptor saccharolyticus* DSM 8903 of the four possible. Whether this distribution of Groups is ultimately representative among all species of the microbial world, it is the representation in the genomes determined to date with many organisms yet to be sequenced.

The evolutionary history of the paralogous nitrogenase family has been extensively studied and branch points have been proposed leading to various designations of protein groups, some with different structures, cofactors, and metabolic function [27]–[29], [43]. Our six groups overlap several of these earlier classifications but our study was restricted to probable or known nitrogenase α-and β-subunits. Because we started from the perspective that sequence alignment should lead to identification of critical residues, our selection of species for inclusion was based on established diversity of phyla and ecological niches without prior knowledge to which nitrogenase protein group a species would belong. Hence, we have made no attempt to organize these groups as branches in their evolutionary history. However, using the accepted 16s-rRNA tree for our chosen species (Figure S1) or the tree based upon the whole proteome similarity (Figure 1), the distribution of our six nitrogenase groups among phyla becomes evident. Although individual groups tend to be more frequently represented in certain classes and phyla, e.g., cyanobacteria have exclusively Group I proteins, Clostridia is notable in having representatives of five of the six groups suggesting horizontal gene transfer has occurred in several stages. Likewise, our Group III proteins, which fall into the “uncharacterized” category in some classifications [28], [29], [43] appear to be distributed across four separated phyla in Figure 1.

The recent work of Dos Santos et al. [33] significantly improves our understanding of the groups by identifying the documented nitrogen fixing species. Dos Santos et al. also proposed that potential nitrogen fixation species should have as a minimum, *nifH*, *nifD*, *nifK*, *nifE*, *nifN*, and *nifB* genes and they provided a second list of probable nitrogen fixing organisms on this basis [33]. In their study, they found a small set of organisms containing clear orthologs of *nifH*, *nifD*, and *nifK* but lacking one or more of the other genes; this group they named “C” and questioned whether they would be nitrogen fixers. Interestingly, as shown in Table S5, many species of their Group C fell in our Groups III and IV, which were assembled entirely by multiple sequence alignment without prior knowledge of other *nif* genes. Indeed, when subsequently investigated, some species of our Group III have both *nifE* and *nifN* and others are missing *nifN*; our Group IV species are missing both *nifE* and *nifN*.

Should species with *nifH*, *nifD* and *nifK* but lacking other *nif* genes be included in the analysis of residues critical to nitrogenase structure-function? It has been suggested that some of these NifD/K proteins might have other enzymatic functions and contain other co-enzymes [28], [29]. Nevertheless, it seems premature to draw definitive conclusions. For example, at least one Group III organism, *Methanocaldococcus sp. FS406-22*, is missing *nifN*, yet it is well documented as a nitrogen fixer by N^15^ incorporation [44]. NifD and NifK alignment in Groups III and IV show these polypeptides are clearly homologous to each other and to those of the other Nif, Anf and Vnf groups. Some but not all members of Group III are missing one or more of the ancillary genes, Table S5 (also see footnote 1). However, based upon sequence differences, it would be difficult to identify which of Group III or IV proteins represent conventional nitrogenases and which might have a different type of functional cofactor and activity.

Most importantly, the NifD sequences from NifN deficient species retain identical residues in the cofactor pocket as found in the known nitrogen fixing species; hence, the insertion of alternate coenzymes seems less likely (see Table S5 and below for discussion of the pocket residues). In our BLAST survey of Groups III and IV for the ancillary genes, as shown in Table S5, the best fit (by bit number) for either NifE or NifN frequently was NifD or NifK. Indeed, in two species having authentic NifE, the better fit, nevertheless, was NifD. In the same way, NifN probes produced good matches for NifK in all Group III and IV species. This close similarity of NifD with NifE and NifK with NifN may not be so surprising because the cofactor synthesis proteins, NifE/N, likely arose by gene duplication of the primordial structural proteins [27]. Thus, it may be that Group III species deficient in NifN can synthesize cofactor by substituting NifK as partner with NifE. Alternatively, the cofactor may be synthesized directly on the NifD/K tetramer without the intervening use of NifE/N, as presumably it occurred in the primordial proteins and, perhaps, in present day Group IV species.

In summary, the genetic analysis defined by Dos Santos et al. [33] is a good initial test for putative nitrogen fixation; nevertheless, the ultimate test is incorporation of N^15^ from N_2_. Likewise, a contrary possibility also needs to be considered: the inability to detect N^15^ incorporation may be the result of failure to reproduce in the laboratory the ecological niches of putative nitrogen fixing organisms. For example, an organism in an obligate consortium, with unknown metabolic constrains, unknown metal requirements, and slow growth rates may not have sufficient N^15^ incorporation to demonstrate nitrogen fixation without using more refined detection methods on single cells [45]. Hence, in our determination of invariant residues, we retain Groups III and IV as potential nitrogen fixing organisms awaiting definitive evidence for each species.

### Conservation of amino acids as strong motifs

The segregation of the nitrogenase proteins into groups is confirmed when the invariant amino acids in the sequences are examined. Beyond the universal invariant residues for all six groups, two other, more limited types of amino acid conservation are considered: residues invariant between groups, and a second more limited designation, residues uniquely invariant in a single group. In the first category residues invariant within a group are also invariant in at least one other group. When pairs of groups are considered, additional invariant residues imply a level of commonality in the evolutionary structure-function between the two groups; the larger the number of common invariant residues between two groups, the more closely these groups are likely to have shared a common evolutionary history constrained by function. The results are given in Tables 2 and 3 for the universally aligned sequences of the α- and β- subunits. In the α-subunit (excluding group specific insertions/deletions), there are 144 invariant residues in Group I and 110 invariant residues in Group II of which 71 residues are co-invariant between the two Groups. Considering the relative number of sequences, Group I (45 sequences/144 invariant) is more conserved than Group II (18 sequences/110 invariant) or Group III (8 sequences/120 invariant). The segregation of Groups I, II, III, and IV is readily justified by the relatively small extent of invariance between groups (beyond the universally invariant residues) and no two groups appear to be more closely related (based upon invariance) than any other two groups. In contrast, Anf and Vnf Groups, encoded by different genes, are more similar to each other (159 common invariant residues) than are any of the *nif* gene derived groups. This is consistent with proposed evolutionary history of the three genes sets [28]–[30]. Indeed, the α-subunit of Group IV is the Nif group closest related to either the Anf or the Vnf Group in terms of the number of co-invariant residues. A similar pattern is observed for the Group IV β-subunit (Table 3) although the number of co-invariant residues is small.

**Table 2:** Invariant Residues, α-Subunit, Common Between Groups.

# Sequences	Group	I	II	III*	IV	Anf	Vnf
45	I	144	71	73	93	68	72
18	II		110	59	84	70	68
8	III*			120	105	78	85
3	IV				359	131	138
12	Anf					256	159
9	Vnf						246

* Group III includes Sec as invariant with Cys.

**Table 3:** Invariant Residues, β-Subunit, Common Between Groups.

# Sequences	Group	I	II	III	IV	Anf	Vnf
45	I	70	44	46	54	44	47
18	II		85	48	67	56	58
8	III			96	72	56	67
3	IV				328	97	103
12	Anf					198	128
9	Vnf						171

The second approach for comparison of the Groups is residue conservation based upon “strong motifs” Bickel et al. [46] defined a strong motif as a group of residues that for a subset of sequences are invariant and never found at those sites in the other homologous sequences. The algorithm was applied to a set of NifD sequences by Glazer and Kechris [30] and α-444 was found to be tryptophan in one subset and tyrosine in all other sequences. On this basis, they identified two categories of nitrogenase. In contrast, we start with already identified subsets (the six groups) and determine which residues are uniquely invariant and never found in the same positions in another group; these are the group specific, strong motifs. This method can be expanded to determine uniquely invariant residues common to any combination of groups. The results of our analysis are given in Tables 2, 3, 4, 5 and Tables S6, S7. For example, there are nine sites where the amino acid is invariant in the Group I α-subunit and there is some other residue in the remaining sequences (Table 4). Indeed, one of these is the previously identified α-Trp444; hence our Group I is equivalent to the Glazer and Kechris [30] α-Trp444 group. Although the number of strong motif residues is not large in the α-subunit, strong motifs are nearly non-existent in the β-subunit with the exception of Group IV (Table 5). The strong motifs to some degree reflect the similarity or diversity within a group and serve to distinguish further between groups; Group I (9 strong motif residues/45 sequences) appears more homogeneous than Group III (only 2 strong motif residues/8 sequences). The strong motifs also may reflect unique properties which justify the separation into groups. The invariant strong motif residues fall into three types: the site is hyper-variable in the other groups, e.g., Group II strong motif residue α-Pro144, nevertheless, has 13 variants in the 95 sequences; the site is a single variant with respect to the other groups, e.g., residue α-Trp 444 in Group I and α-Tyr 444 in all others; or the site is a strong motif in most groups, e.g., α-Leu/Ala/Met/Gly193. The large number of residues constituting the strong motif for Group IV likely reflects the small number of sequences in the group and the close phylogeny of the group species. Nevertheless, it is remarkable that ca 10% of the residue sites in Group IV NifD are group invariant and ***never*** found in any of the other 92 sequences.

**Table 4:** Number of Strong Motif Residues, α-Subunit.

# Sequences	Group	I	II	III	IV	Anf	Vnf
45	I	9	5	0	1	0	0
18	II		7	0	0	1	0
8	III			2	1	0	0
3	IV				32	0	2
12	Anf					23	15
9	Vnf						15

**Table 5:** Number of Strong Motif Residues, β-Subunit.

# Sequences	Group	I	II	III	IV	Anf	Vnf
45	I	3	0	0	0	0	0
18	II		2	0	0	0	0
8	III			1	1	0	0
3	IV				30	0	0
12	Anf					3	6
9	Vnf						1

Perhaps the most significant consequence of the strong motif concept is the ability to place a new sequence in a group (Tables S6 and S7). The present analysis greatly expands the utility to identify the gene of origin for a nitrogenase. Many of the strong motif amino acid sites are limited to a single group although several are more universal. Residue α-69 readily distinguishes *nif*, *anf*, or *vnf* genetic origin by the significantly different residues glycine, histidine, or leucine at this position. Five sites across the two subunits are unique to *nif* origin: namely, α-Ala65, α-Gly69, α-Tyr387, β-Arg105 and β-Pro144 are unique to Nif D and NifK. Proteins of *anf* or *vnf* origin are distinguished from each other by unique amino acids at α-274, α-364, α-390, α-394 α-427, and α-451 where each group has a strong motif (Table S6). Whether these strong motif residues are of functional significance is not evident but they do provide a means to identify the genetic origin of a given protein. With the caveat expressed above that new sequences may reduce the number of conserved residues, identification of a gene of origin (group specific identification) is not dependent on a single site but rather on the ensemble of residues. The utility of the strong motifs was evident in several situations during the building of our data base. For example, the protein identified by sequence accession CCD03004.1 is annotated as “nitrogenase molybdenum-iron protein alpha chain, nifD [*Azospirillum brasilense* Sp245]” yet a survey of the strong motifs quickly identified it as Vnf not Nif. Hence, this sequence was placed as a member of the Vnf group in our data base as V-02 (Table S1). To date *vnf* and *anf* genotypes have occurred only as “alternate” or secondary to *nif*, yet presumably either could be found as the sole nitrogenase gene. The strong residues and sequence alignment should readily place the genotype of new nitrogenase proteins with the potential to identify a sole nitrogenase gene type as one of the designated alternate forms.

### Seleno-cysteine (Sec) containing NifD, α-subunit

The strict rules of identifying a residue as invariant were abrogated in one situation. *Caldicellulosiruptor saccharolyticus* NifD BLAST analysis indicated two of the related sequences, those from *Candidatus Desulforudis audaxviator* and *Thermodesulfatator indicus,* contained seleno-cysteine at position α-62, an invariant P-cluster ligand (*A. vinelandii* numbering). A 22 residue peptide that overlapped the Sec position and was sufficiently specific to produce only α-subunit homologues, was used in a BLAST search (500 sequence cutoff) of the full translated NCBI DNA data repository. Only one additional Nif sequence containing Sec was found, NifD in *Desulfotomaculum kuznetsovii*, and, with periodical re-testing of the data base, no new sequences have been discovered. All three Sec containing sequences belonged to Group III in terms of insertion/deletion patterns, strong motif, and invariant residues for both the α- and β- subunits. All three species are lacking *nifN*, and none have been proven to be nitrogen fixing by N^15^ incorporation, Table S5. The probable identification of α-Sec62 was verified by established criteria: the amber stop codon, TAG, in the appropriate DNA reading frame; the presence of genes for Sel A (selenocysteine synthase), Sel B (selenocysteine-specific translation elongation factor), and Sel D (selenophosphate synthase); and most importantly, the stem-loop signature bSECIS in the mRNA [47], [48]. All conditions were met for these three species, hence, Cys along with Sec are considered as invariant residue 62. Curiously, other species in Group III, as well as members of other groups, contain components of the necessary machinery for Sec insertion without exploiting them for their nitrogenase.

No other putative Sec residues were found in the NifD, NifK or NifH from these three species which leads to speculation as to what role this highly specific substitution might have. For example, Sec is usually found as part of an enzyme's active site whereas in these nitrogenases, α-Sec62 (*A. vinelandii* numbering) is a putative ligand to the electron transfer P-cluster [49]–[51]. Sec has a significantly lower pK_a_ than Cys leading to higher nucleophilicity for Sec at neutral pH [52], yet selenium terminal ligands to Fe:S clusters do not have appreciable effects on the redox potential of at least two oxidation states in model compounds [53]. Hence, extrapolation of these Sec properties to the P-cluster at the functioning pH and temperature for Sec-containing nitrogenase would be tenuous. In the active site of one class of hydrogenases, Sec enables rapid recovery from oxygen inactivation [54]. Such a function for α-Sec62 seems unlikely as the species with the Sec containing NifD are strict anaerobes, but this does not preclude some other unique function for a Sec radical. Another possibility involves the nature of the P-cluster. The presumption is that the nitrogenase P-clusters are always Fe:S based, yet an Fe:Se P-cluster cannot be excluded which might require a Sec ligand. Interestingly in this regard, α-Sec62 is covariant with β-Ala92; all other sequences have β-Gly/Ser92. The β carbon of β-Ser92 (presumably occupied by the analogous β carbon of Ala) is in van der Waals contact with a P-cluster sulfur at the same face as the iron ligand α-Cys62. Finally, an alternate role for the Sec could be at the level of protein expression. In other systems, the protein synthesis rate is controlled by restrictive, low population codons positioned early in the mRNA sequence [55]. In Group III, NifD is shorter than Group I; hence, in Group III sequences, Sec is residue 46 from the amino terminal (residue 62 in the universal numbering based upon the *A. vinelandii* NifD) and in this position, with the unusual codon and the associated required stem-loop bSECIS mRNA fold, Sec incorporation could serve to regulate the NifD synthesis.

### Multiple sequence alignment and evaluation of metal binding sites

As the centers for electron transfer and substrate reduction, the P-cluster and the cofactor are dominant features in the structure-function of nitrogenase (see Figure 1). An early goal for the multiple sequence alignment was to identify core residues in the environments of these metal centers that might influence their properties. A further goal was to correlate any residue variance with substrate and product differences associated with the cofactor depending on whether it contains a Mo, V, or Fe atom at the variable position. Indeed, residues in the cofactor pocket have been altered by mutagenesis with the objective of altering the substrate specificity (see e.g., [56]–[58]). Using the 1.16 Å resolution *A. vinelandii* crystal structure, all residues within 5 Å of the P-cluster or cofactor including both the metal cluster and homocitric acid were identified and the variants were compiled from the multi-sequence alignment. The results are given in Tables S8, S9, and S10.

Fifteen residues from the α-subunit and 13 residues from the β-subunit define the cavity for the P-cluster which serves as the electron transfer center between the Fe-protein and the cofactor substrate reduction center. Only 11 residues are invariant: the six cysteinyl ligands and five residues (Gly or Pro) that appear to direct the ligand backbone geometry. Because the P-cluster bridges the two subunits, many of the residues in the P-cluster cavity compose the α-β subunit interface; yet, the variation in these residues indicates the interface and pocket around the cluster is diverse in detail. Indeed, as shown in Table S8, no simple correlation was evident between amino acid residues in the P-cluster environment and the six classes of nitrogenase that might explain differences in substrate specificity between groups. This is remarkable for a cluster that seemingly must be controlled for redox potential, oxidation state, and gated electron transfer in order to function in the full nitrogenase turnover.

**Figure 4:**
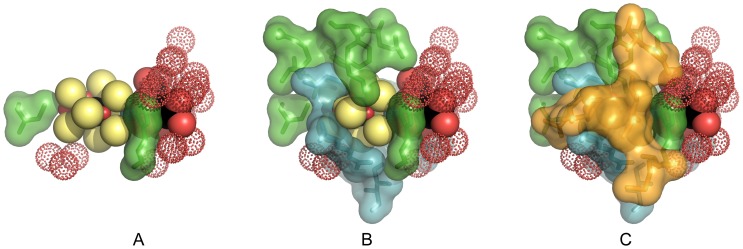
Cofactor environment showing amino acid residues at 5 Å contact. Cofactor including homocitric acid, α-His442, and α-Cys 275 ligands are shown as CPK spheres. Waters are red dot spheres. Dark green surface and sticks represent invariant residues. Light teal surface and sticks represent single variant residues. Bright orange surface and sticks represent multiple variant residues. (See Tables S9 and S10) A. Cofactor with α-Cys275 and α-His442 ligands. B. Invariant and single variant residues added. C Multiple variant residues added. Water sheet between homocitric acid and β-subunit is on the right. Amino acids that interact only by H-bond through water atoms are omitted. Figure was prepared using 3U7Q.pdb and Pymol (http://pymol.org/).

The cofactor environment can be divided into two parts determined by areas around the metal cluster or around the homocitric acid portions. The cluster environment appears to be more highly conserved as indicated in Table S9, where 14 of 19 residues across all six groups are invariant (9) or highly similar, single variant (5) residues. Within each of the six Groups, the residues around the cluster have a higher degree of conservation–higher fraction of invariant residues–than for the full 95 sequences. However, most significantly, there does not appear to be any obvious correlation of amino acid variants to the gene of origin (*nif*, *anf*, or *vnf*) or to the absence of the ancillary NifE/N proteins (see discussion above). A detailed structural analysis revealed that the most highly variable residues are not randomly distributed around the cofactor metal cluster but are concentrated on one face as shown in Figure 4. This face containing the hyper-variable residues is towards, though not on, the surface of the protein, e.g., variable α-Leu-358 is partially exposed to solvent prior to cofactor insertion [59]. The highly conserved, invariant and single variant residues on the other faces are directed towards the P-cluster. Several of these residues previously have been probed by site specific mutagenesis and have been shown to alter the cofactor spectral properties and substrate specificity, e.g., α-Val70, α-Arg96, and α-His195 [56], [57] which further emphasizes the importance of the conserved residues around the cofactor in substrate binding and electron transfer.

The 5 Å limit for the homocitric acid environment extends to the α-β-subunit interface and includes three β-subunit residues. However, these three residues along with five residues of the α-subunit do not make direct contact with the homocitric acid but are separated by a water layer along the interface and contact the homocitric acid by H-bonds through the water atoms (Table S10). This water pool has been previously described and postulated to be part of an H-bonded proton relay for substrate reduction [60]–[62]. Of the 14 residues making direct or indirect, water-mediated contact with the homocitric acid, only three are invariant and two of these, α-Gln191 and α-His442 are also residues associated with the cofactor cluster.

Component I contains a third metal site, ostensibly to stabilize the interface of the two β-subunits. By symmetry there are two identical mononuclear metal sites with half the ligands from each β-subunit. The ligands are the highly conserved carboxyl side chains of β-Asp353 and β-Asp357 from one β-subunit of the pair with the peptide backbone carbonyl of β-108 and the carboxyl side chain of β-Glu-109 of the second β-subunit (See Table S4). Although none of the coordinating side chain residues are invariant, the variants are minor and also could serve as ligands; Asn for Asp and Asp for Glu. Likewise, β-108 is either Arg or Lys with a single outlier variant, Gln.

The three alternative nitrogen fixing proteins were initially found to have related but different cofactors containing either molybdenum, vanadium, or iron only [25]. Which specific structural protein was expressed and which cofactor was synthesized was controlled either directly or indirectly by the metals available. However, each of the three types of cofactor were found to be compatible with each of the three precursor apo-proteins, encoded by their cognate genes, albeit with modified enzymological properties commensurate with both the protein and cofactor of origin [25]. Hence, it has been a central question to distinguish the relative roles of the protein and the cofactor metal in determining function. Recently, McGlynn et al. [43] proposed that the metal dependence of uncharacterized nitrogenases could be determined from characteristic amino acid residues and phylogenetic clustering of D gene homologues. In their evaluation of the Archaeal ANME-2 protein, they used the α-subunit residue positions α-65, α-69, α-96, and α-380 to assign the protein as FeMoco based. As expected, these residues are in our analysis and we confirm that the D gene was *nif* derived and a member of Group III. However, caution is advised for the interpretation of the cofactor and associated metal content. Namely, amino acids immediately around the cofactor metal sites do not directly correlate to cofactor type. Furthermore, the Anf and Vnf groups should be treated separately as their cofactors are as distinct from each other in expressed substrate profile as either is from that of the Nif groups [25]. Rather, what can be said is that a new nitrogenase can be confidently placed in one of the six protein groups by general sequence homology augmented by the strong motifs. This assignment, however, indicates the gene of origin not the metal content of the cofactor.

Genetic analysis is only a guide to the phenotype. The critical test of the metal content must be direct chemical analysis of the isolated protein which is not a trivial undertaking for the protein from many species. Because the cofactor synthesis is under a variety of cellular metabolic controls including metal transport, the metal that is incorporated in the cofactor is sensitive to multiple factors beyond that of which structural protein is expressed. For example, with the proper genetic manipulation of the molybdenum regulation, FeMoco can be synthesized and inserted in AnfD/K [63]. Likewise, tungsten (presumably replacing molybdenum) has been incorporated in nitrogenase when the organism was genetically and metabolically manipulated, albeit the tungsten containing enzyme is no longer capable of dinitrogen reduction but does retain high proton reduction activity [64]. Thus, the nitrogenase gene that is harbored or expressed by an organism, especially organisms from ecological niches less well understood, may not fall into the traditional correlation that FeMoco is equivalent to *nif* genes.

## Conclusions and Summary

Multiple amino acid sequence alignment of the α- and β- subunits for the three nitrogenase genotypes is a powerful tool to evaluate protein structure-function properties and natural history. Because the sequences were chosen from species from diverse ecological and phylogenetic sources, residues retained as invariant and single variant by natural selection are deemed the critical core. The small number of core residues (ca. 17%) encompasses all three genotypes and emphasizes the homology of the three groups. The *nif* genotype can be subdivided into four groups based on insertion, deletion, extension, and homology differences in the sequences. The *vnf* and *anf* genotypes represent two additional groups. Each of the six groups exhibits a small number of residues that are uniquely invariant within the group. Hence, these unique (strong motif) residues serve to identify the group and genotype for a newly sequenced species.

One consequence of the multiple sequence alignment was the identification of our Group III that overlaps with previously catalogued species as either “uncharacterized nitrogen fixers”, potential nitrogen fixers, or non-nitrogen fixing paralogues [28], [29], [33]. Although the co-linearity of the sequences for both the α- and β-subunits independently catalogue members of Group III, nevertheless, the member species are quite diverse in other respects. The group has a known nitrogen fixing member lacking one ancillary protein, NifN, usually considered mandatory for functional nitrogenase. Other closely related sequences are from species with a full complement of ancillary proteins. Group III also contains three species where the P-cluster ligand, α-Cys62 is coded as seleno-cysteine that may provide a window on the P-cluster function in the overall nitrogenase mechanism. This group and Group IV clearly indicate the need for direct demonstration of nitrogen fixation by N^15^ incorporation and metal content of the cofactor taking into consideration the special features of the ecological niche for the organism.

Multiple sequence alignment has utility in evaluating the three metal centers in Component 1 proteins. The P-cluster environment was remarkably diverse, with a limited number of conserved residues other than the metal ligands. In contrast, the cofactor pocket was highly conserved with little indication of group specificity related to metal type in the enclosed cofactor. Most interesting, the ca.25% of the pocket residues are multi-variable and are located on one side of the cofactor, away from the other functional regions of the α-subunit which emphasizes the strict retention of the other residues. Although strong motifs can serve to identify the gene of origin, prudence is strongly suggested when attempting to deduce the cofactor metal content from sequence analysis.

It is beyond the scope of this study to evaluate the extensive and insightful literature on site specific mutagenesis directed to understanding the role and environment of individual residues in the nitrogenase function. However, it should be noted that natural selection has provided a substantial catalogue of required as well as allowed functional variation for each residue in the sequence. The multi-sequence alignment as analyzed in the tables presented here coupled to the very high resolution structures now available allows the further consideration of earlier mutagenesis results and interpretations. Our study is directed to the evaluation of the sequence conservation in terms of structure-function analysis ultimately using the three-dimensional protein structure.

## Supporting Information

Figure S1 Phylogeny of species and groups based on 16S rRNA. Species identifiers (abbreviated from Table S1) are for the six nitrogenase groups; species with both Nif and either Anf or Vnf have more than one identifier. For three species, strains were used that were different than used for the NifD/K alignment. They are: I-24-*Methylocystis sp*. (gi:402770565), I-36-*Scytonema sp* (gi: 319748277), and II-07-*Clostridium pasteurianum* (gi:270265548).
(PDF)

Table S1 Identification of Species, Lineage, and Gene ID used for Multiple Sequence Alignment.
(PDF)

Table S2 Residues co-aligned across the 95 sequences.
(PDF)

Table S3 Amino Acid Residue Variance in α-Subunit (Gene D).
(PDF)

Table S4 Amino Acid Residue Variance in β-Subunit (Gene K).
(PDF)

Table S5 Properties of Nif genes in Groups III and IV.
(PDF)

Table S6 Strong Motifs in Core Alignment α-subunit (Gene D).
(PDF)

Table S7 Strong Motifs in Core Alignment β-subunit (Gene K).
(PDF)

Table S8 α, β-Subunit Residues within 5 Å any Atom in P-cluster.
(PDF)

Table S9 Residues in α-Subunit within 5 Å of Any Atom of Metal Cluster Component of FeMoco.
(PDF)

Table S10 Residues Within 5 Å of Any Atom of Homocitric Acid Component of FeMoco.
(PDF)
