# Supplementary figures and images for: Multiple Amino Acid Sequence Alignment Nitrogenase Component 1: Insights into Phylogenetics and Structure-Function Relationships
Source: PLoS One. 2013 Sep 3;8(9):e72751. doi: 10.1371/journal.pone.0072751 (PMC3760896; doi:10.1371/journal.pone.0072751)

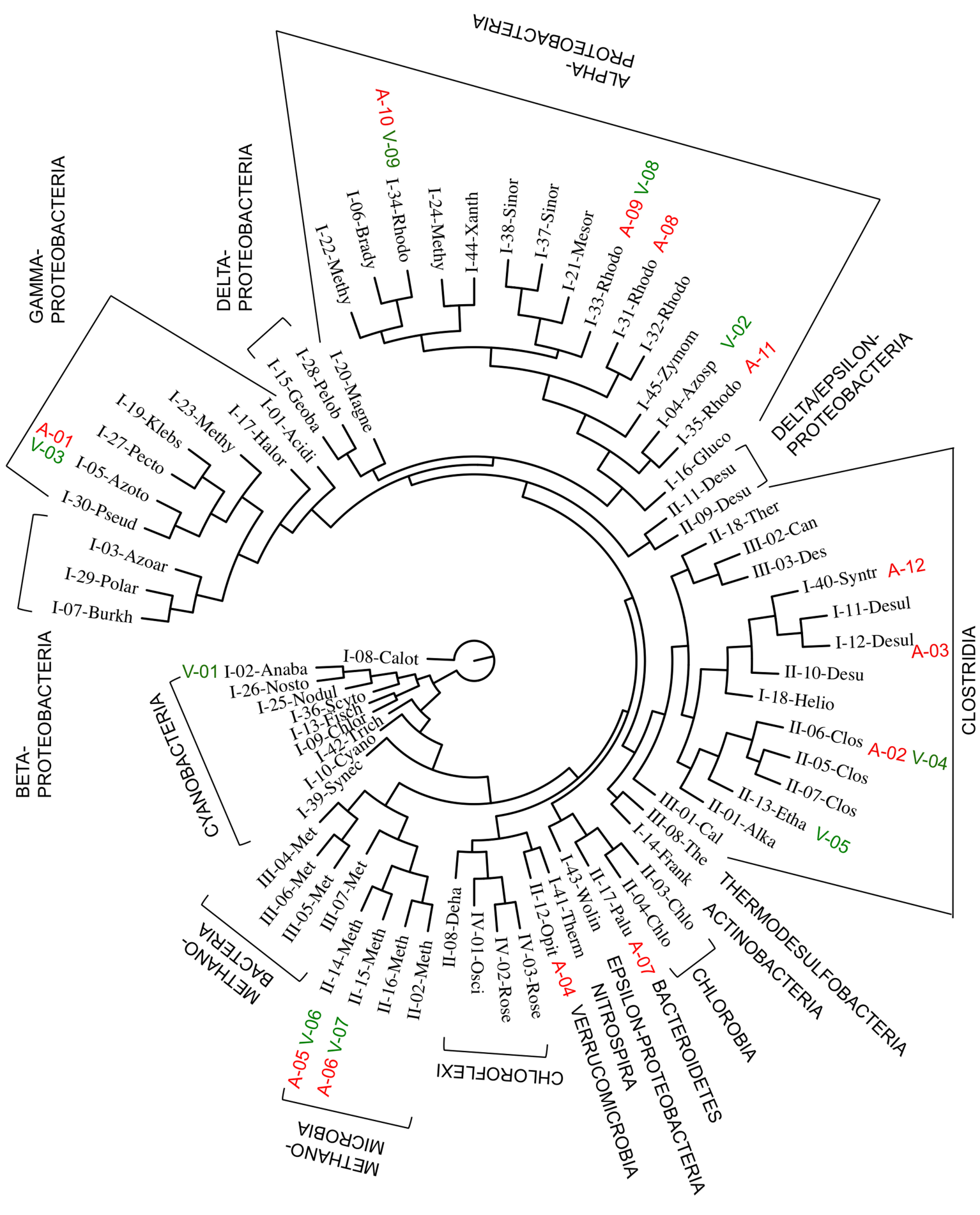

Supplement: Figure S1 — Phylogeny of species and groups based on 16S rRNA. Species identifiers (abbreviated from Table S1) are for the six nitrogenase groups; species with both Nif and either Anf or Vnf have more than one identifier. For three species, strains were used that were different than used for the NifD/K alignment. They are: I-24-Methylocystis sp. (gi:402770565), I-36-Scytonema sp (gi: 319748277), and II-07-Clostridium pasteurianum (gi:270265548). (PDF) [file pone.0072751.s001.pdf]
