# Supplementary material for: Multiple Amino Acid Sequence Alignment Nitrogenase Component 1: Insights into Phylogenetics and Structure-Function Relationships
Source: PLoS One. 2013 Sep 3;8(9):e72751. doi: 10.1371/journal.pone.0072751 (PMC3760896; doi:10.1371/journal.pone.0072751)
Supplement: Table S1 — Identification of Species, Lineage, and Gene ID used for Multiple Sequence Alignment. (PDF) [file pone.0072751.s002.pdf]

Table S-1. Identification of Species, Lineage, and Gene ID used for Multiple Sequence Alignment
All Sequences are from genomes unless #

**Nif Group I**	**D-Gene, ID**	**K-Gene, ID**	**Lineage**
I-01 Acidithiobacillus ferrooxidans ATCC 23270	YP_002425949.1	YP_002425948.1	Gammaproteobacteria
I-02 Anabaena variabilis ATCC 29413	YP_324742.1	YP_324743.1	Cyanobacteria
I-03 Azoarcus sp. BH72	YP_932043.1	YP_932044.1	Betaproteobacteria
I-04 Azospirillum brasilense Sp245	CCC97491.1	CCC97490.1	Alphaproteobacteria
I-05 Azotobacter vinelandii DJ	YP_002797379.1	YP_002797380.1	Gammaproteobacteria
I-06 Bradyrhizobium japonicum	AAG60729.1	AAG60730.1	Alphaproteobacteria
I-07 Burkholderia xenovorans LB400	YP_553848.1	YP_553847.1	Betaproteobacteria
I-08 Calothrix desertica PCC 7102 #	AAN63662.1	ACA61792.1	Cyanobacteria
I-09 Chlorogloeopsis fritschii PCC 6912 #	AAP40828.1	ACA61793.1	Cyanobateria
I-10 Cyanothece sp. ATCC 51142	AAB61283.1	AAB61284.1	Cyanobacteria
I-11 Desulfitobacterium hafniense DCB-2	YP_002457546.1	YP_002457547.1	Clostridia
I-12 Desulfosporosinus sp. OT	ZP_08811362.1	ZP_08811361.1	Clostridia
I-13 Fischerella muscicola PCC 7414 #	AAN63673.1	ACA61795.1	Cyanobacteria
I-14 Frankia alni ACN14a	YP_716938.1	YP_716937.1	Actinobacteria
I-15 Geobacter sulfurreducens PCA	NP_953864.1	|NP_953863.1	Deltaproteobacteria
I-16 Gluconacetobacter diazotrophicus PAl 5	YP_001600721.1	YP_001600722.1	Alphaproteobacteria
I-17 Halorhodospira halophila SL1	YP_001001869.1	YP_001001868.1	Gammaproteobacteria
I-18 Heliobacterium modesticaldum Ice1	YP_001679707.1	YP_001679708.1	Clostridia
I-19 Klebsiella pneumoniae 342	YP_002237564.1	YP_002237563.1	Gammaproteobacteria
I-20 Magnetococcus marinus MC-1	YP_865118.1	YP_865117.1	Alphaproteobacteria
I-21 Mesorhizobium loti MAFF303099	BAB52276.1	BAB52277.1	Alphaproteobacteria
I-22 Methylobacterium sp. 4-46	YP_001770352.1	YP_001770353.1	Alphaproteobacteria
I-23 Methylococcus capsulatus str. Bath	YP_112765.1	|YP_112766.1	Gammaproteobacteria
I-24 Methylocystis sp. ATCC 49242	ZP_08073551.1	ZP_08073550.1	Alphaproteobacteria
I-25 Nodularia spumigena CCY9414	ZP_01628430.1	ZP_01628425.1	Cyanobacteria
I-26 Nostoc sp. PCC 7120	NP_485484.1	NP_485483.1	Cyanobacteria
I-27 Pectobacterium atrosepticum SCRI1043	YP_051046.1	YP_051045.1	Gammaproteobacteria
I-28 Pelobacter carbinolicus DSM 2380	YP_357509.1	YP_357510.1	Deltaproteobacteria
I-29 Polaromonas naphthalenivorans CJ2	YP_982572.1	YP_982571.1	Betaproteobacteria
I-30 Pseudomonas stutzeri A1501	YP_001171864.1	YP_001171865.1	Gammaproteobacteria
I-31 Rhodobacter capsulatus SB 1003	YP_003576743.1	YP_003576742.1	Alphaproteobacteria
I-32 Rhodobacter sphaeroides 2.4.1	YP_353613.1	YP_353612.1	Alphaproteobacteria
I-33 Rhodomicrobium vannielii ATCC 17100	YP_004012289.1	YP_004012290.1	Alphaproteobacteria
I-34 Rhodopseudomonas palustris CGA009	CAE30059.1	CAE30058.1	Alphaproteobacteria
I-35 Rhodospirillum rubrum ATCC 11170	YP_426099.1	YP_426100.1	Alphaproteobacteria
I-36 Scytonema sp. PCC 7814 #	AAP40826.1	ACA61802.1	Cyanobacteria
I-37 Sinorhizobium fredii NGR234]	NP_444137.1	NP_444138.1	Alphaproteobacteria
I-38 Sinorhizobium medicae WSM419	YP_001314761.1	YP_001314760.1	Alphaproteobacteria
I-39 Synechococcus sp. JA-2-3B'a(2-13)	YP_476681.1	YP_476682.1	Cyanobacteria
I-40 Syntrophobotulus glycolicus DSM 8271	YP_004267111.1	YP_004267110.1	Clostridia
I-41 Thermodesulfovibrio yellowstonii DSM 11347	ACI21314.1	ACI20660.1|	Nitrospira
I-42 Trichodesmium erythraeum IMS101	AAF82638.1	AAF82639.1	Cyanobacteria
I-43 Wolinella succinogenes	CAE10458.1	CAE10457.1	Epsilonproteobacteria
I-44 Xanthobacter autotrophicus Py2	YP_001415005.1	YP_001415006.1	Alphaproteobacteria
I-45 Zymomonas mobilis subsp. mobilis ZM4	YP_163559.1	YP_163560.1	Alphaproteobacteria

Nif Group II	D-Gene, ID	K-Gene, ID	Lineage
II-01 Alkaliphilus metalliredigens QYMF	YP_001321307.1	YP_001321306.1	Clostridia
II-02 Candidatus Methanoregula boonei 6A8	YP_001404306.1	YP_001404307.1	Methanomicrobia
II-03 Chlorobium phaeobacteroides DSM 266	ABL64794.1	ABL64793.1	Chlorobia
II-04 Chlorobium tepidum TLS	NP_662420.1	NP_662421.1	Chlorobia
II-05 Clostridium beijerinckii NCIMB 8052	YP_001309128.1	YP_001309129.1	Clostridia
II-06 Clostridium kluyveri DSM 555	EDK35086.1	EDK35085.1	Clostridia
II-07 Clostridium pasteurianum DSM 525	WP_003447876.1	WP_003447875.1	Clostridia
II-08 Dehalococcoides ethenogenes 195	YP_181869.1	YP_181868.1	Chloroflexi
II-09 Desulfonatronospira thiodismutans ASO3-1	ZP_07015346.1	ZP_07015347.1	Chloroflexi
II-10 Desulfotomaculum reducens MI-01	YP_001114147.1	YP_001114146.1	Clostridia
II-11 Desulfovibrio vulgaris str. Hildenborough	YP_009052.1	YP_009051.1	Deltaproteobacteria
II-12 Diplosphaera colitermitum TAV5	ZP_09596373.1	ZP_09596374.1	Verrucomicrobia
II-13 Ethanoligenens harbinense YUAN-3	YP_004091831.1	YP_004091830.1	Clostridia
II-14 Methanosarcina acetivorans C2A	NP_618769.1	NP_618770.1	Methanomicrobia
II-15 Methanosarcina barkeri	YP_303733.1	YP_303732.1	Methanomicrobia
II-16 Methanosphaerula palustris E1-9c	ACL15931.1	ACL15930.1	Methanomicrobia
II-17 Paludibacter propionicigenes WB4	YP_004042557.1	YP_004042558.1	Bacteroidetes
II-18 Thermoanaerobacterium thermosaccharolyticum DSM 571	ADL69354.1	ADL69353.1	Clostridia

Nif Group III	D-Gene, ID	K-Gene, ID	Lineage
III-01 Caldicellulosiruptor saccharolyticus DSM 8903	YP_001181231.1	YP_001181230.1	Clostridia
III-02 Candidatus Desulforudis audaxviator MP104C	YP_001716346.1	YP_001716347.1	Clostridia
III-03 Desulfotomaculum kuznetsovii DSM 6115	YP_004518628.1	YP_004518627.1	Clostridia
III-04 Methanocaldococcus sp. FS406-22	YP_003457471.1	YP_003457472.1	Methanococci
III-05 Methanococcus aeolicus Nankai-03	ABR57007.1	ABR57006.1	Methanococci
III-06 Methanococcus maripaludis C5	YP_001097187.1	YP_001097188.1	Methanococci
III-07 Methanothermobacter thermautotrophicus, Delta H	NP_276676.1	NP_276677.1	Methanobacteria
III-08 Thermodesulfatator indicus DSM 15286	YP_004625888.1	YP_004625889.1	Thermodesulfobacteria

Nif Group IV	D-Gene, ID	K-Gene, ID	Lineage
IV-01 Oscillochloris trichoides DG6	ZP_07684112.1	ZP_07684111.1	Methanococci
IV-02 Roseiflexus sp. RS-1	YP_001275556.1	YP_001275555.1	Chloroflexi
IV-03 Roseiflexus castenholzii DSM 13941	YP_001434092.1	YP_001434091.1	Chloroflexi

Group Anf	D-Gene, ID	K-Gene, ID	Lineage
A-01 Azotobacter vinelandii DJ	YP_002801974.1	YP_002801972.1	Gammaproteobacteria
A-02 Clostridium kluyveri DSM 555	YP_001393772.1	YP_001393774.1	Clostridia
A-03 Desulfosporosinus sp. OT	ZP_08813146.1	ZP_08813148.1	Clostridia
A-04 Diplosphaera colitermitum TAV2	ZP_03727075.1	ZP_03727077.1	Verrucomicrobia
A-05 Methanosarcina acetivorans C2A	NP_616149.1	NP_616147.1	Methanomicrobia
A-06 Methanosarcina barkeri str. Fusaro	AAZ70500.1	AAZ70498.1	Methanomicrobia
A-07 Paludibacter propionicigenes WB4	ADQ79509.1	ADQ79507.1	Bacteroidetes
A-08 Rhodobacter capsulatus SB 1003	ADE84351.1	ADE84353.1	Alphaproteobacteria
A-09 Rhodomicrobium vannielii ATCC 17100	YP_004013646.1	YP_004013644.1	Alphaproteobacteria
A-10 Rhodopseudomonas palustris CGA009	NP_946787.1	NP_946785.1	Alphaproteobacteria
A-11 Rhodospirillum rubrum ATCC 11170	YP_426482.1	YP_426480.1	Alphaproteobacteria
A-12 Syntrophobotulus glycolicus DSM 8271	ADY57120.1	ADY57118.1	Clostridia

Group Vnf	D-Gene, ID	K-Gene, ID	Lineage
V-01 Anabaena variabilis ATCC 29413	ABA23631.1	ABA23632.1|	Cyanobacteria
V-02 Azospirillum brasilense Sp245	CCD03004.1	CCD03006.1	Alphaproteobacteroa
V-03 Azotobacter vinelandii DJ	YP_002797497.1	YP_002797495.1	Gammaproteobacteria
V-04 Clostridium kluyveri DSM 555	YP_001395137.1	YP_001395135.1	Clostridia
V-05 Ethanoligenens harbinense YUAN-3	YP_004092555.1	YP_004092557.1	Clostridia
V-06 Methanosarcina acetivorans C2A	NP_616155.1	NP_616157.1|	Methanomicrobia
V-07 Methanosarcina barkeri str. Fusaro	AAZ71203.1	AAZ71201.1	Methanomicrobia
V-08 Rhodomicrobium vannielii ATCC 17100	YP_004010895.1	YP_004010897.1	Proteobacteria
V-09 Rhodopseudomonas palustris CGA009	NP_946728.1	NP_946730.1	Proteobacteria
