# Supplementary material for: Multiple Amino Acid Sequence Alignment Nitrogenase Component 1: Insights into Phylogenetics and Structure-Function Relationships
Source: PLoS One. 2013 Sep 3;8(9):e72751. doi: 10.1371/journal.pone.0072751 (PMC3760896; doi:10.1371/journal.pone.0072751)
Supplement: Table S2 — Residues co-aligned across the 95 sequences. (PDF) [file pone.0072751.s003.pdf]

Table S-2. Residues co-aligned across the 95 sequences

Gene D (α-subunit)
A. vinelandii sequence numbers
17-35; 48-89; 90-93; 95-100; 108-110; 112-144; 146-174;
176-207; 221-317; 320-368; 369-391; 394-446; 448-479

Block Residues	i, insertions; d, deletions, within blocks		
17-35			
48-100	89-i	94-d	
108-207	111-d	145-d	174-5-i/d
221-391	318-9-i/d	368-i	
394-479	397-i	447-d	

Gene K (β-subunit)
A. vinelandii sequence numbers
Co-aligned residues: 62-142; 143-170; 177-180; 182-210; 225-233; 235-250; 252-257; 259-265; 270-273; 274-298; 300-306; 309; 311-316; 319-357; 358-359; 361-397; 398; 400-410; 420-422; 426-433; 435; 439-448; 449-455; 464-508

Block Residues			i, insertions; d, deletions, within blocks					
62-170	142-i							
177-210	181-i							
225-265	233-4-i/d	251-i/d	258-d					
270-398	273-i	299-d	307-8-d	310-d	317-8-d	357-i	360-d	397-i
400-410								
420-422								
426-435	434-d							
439-455	448-i							
464-508								
