# Supplementary material for: Multiple Amino Acid Sequence Alignment Nitrogenase Component 1: Insights into Phylogenetics and Structure-Function Relationships
Source: PLoS One. 2013 Sep 3;8(9):e72751. doi: 10.1371/journal.pone.0072751 (PMC3760896; doi:10.1371/journal.pone.0072751)
Supplement: Table S3 — Amino Acid Residue Variance in α-Subunit (Gene D). (PDF) [file pone.0072751.s004.pdf]

Table S-3. Amino Acid Residue Variance in α-Subunit (Gene D)[1,2,3]

Residue number	Invariant	Dominant >90%	Single variant, Dominant	Single variant	Double variant, Dominant	Double variant	Multiple variant Dominant
	41	40	21	18	11	19	8
28	R						
31		90 H					H (s,a,q)
49						N/Y/d	
54		94 P	P/a				
55	G						
57						M/I/L	
58				T/S			
60	R						
61	G						
62	C						
64				Y/F			
65				A/C			
66	G						
68				K/R			
69						G/H/L	
70	V						
71				V/I			
74						P/V/a	
77	D						
83	H						
84		90 G			G/a/s		
85	P						
87	G						
88	C						
93						W/R/g	
96				R/K			
97		92 R	R/p				
116				S/T			
117		93 D	D/n				
120		93 E	E/d				
123				I/V			

Table S-3, continued

Residue number	Invariant	Dominant >90%	Single variant, Dominant	Single variant	Double variant, Dominant	Double variant	Multiple variant Dominant
124				V/I			
125				F/Y			
126	G						
127				G/A			
131	L						
137		91 E			E/d/q		
142		89 F					F (m,n,c)
153						T/E/n	
154	C						
157				G/A			
158	L						
159	I						
160	G						
161		94 D	D/g				
162		92 D	D/n				
178				V/I			
183						C/S/A	
184						E/P/a	
185	G						
188	G						
189						V/P/I	
190		93 S	S/t				
191	Q						
192	S						
194	G						
195	H						
196		94 H	H/q				
199		91 N			N/s/c		
227		93G	G,a				
228				D/E			
229		92 Y			Y/f/h		
230	N						
231		91 I	I/m				
233		87 G			G/W/n		

Table S-3, continued

Residue number	Invariant	Dominant >90%	Single variant, Dominant	Single variant	Double variant, Dominant	Double variant	Multiple variant Dominant
234		94 D	D/e				
246	G						
255	G						
256				D/N			
270		91 L					L (f,m,v)
272						L/V/I	
275	C						
277	R						
278	S						
281	Y						
293		91 P					P (a,gr)
301	G						
309		86 L			L/I/m		
310		88 R					R (k,l,y)
313						A/G/c	
316		93 F	F/l				
328	I						
346		86 L			L/C/t		
348		94 G	G/d				
356	G						
357		93 G	G/a				
358						L/S/P	
359				R/K			
361						R/W/H	
362		94 H	H/t				
371		94 G	G/a				
381	F						
383	H						
385						D/G/E	
386		92 D	D/g				
387						Y/F/M	
389						R/K/G	
402	D						
408		94 E	E/d				
418		90 P					P (a,l,v)

Table S-3, continued

Residue number	Invariant	Dominant >90%	Single variant, Dominant	Single variant	Double variant, Dominant	Double variant	Multiple variant Dominant
**419**		**90 D**			**D/s/e**		
**424**	**G**						
**426**				**K/R**			
**428**						**K/G/R**	
**433**	**K**						
**437**		**88 P**					**P (f,l,q,v)**
**442**	**H**						
**444**				**Y/W**			
**445**						**D/H/E**	
**448**	**G**						
**449**		**93 P**	**P/r**				
**450**		**92 Y**			**Y/f/w**		
**452**		**89 G**			**G/A/s**		
**453**						**Y/F/v**	
**455**		**94 G**	**G/r**				
**460**		**87 A**			**A/Y/g**		
**462**		**93 D**	**D/e**				
**470**		**92 P**					**P (k,r,t)**

[1] Based upon core sequence alignment, 95 sequences. α-subunit co-aligned residues: 17-35; 48-89; 90-93; 95-100; 108-110; 112-144; 146-174; 176-207; 221-317; 320-368; 369-391; 394-446; 448-479. *A. vinelandii* , α-subunit numbering.

[2] Residue numbers based upon the *Azotobacter vinelandii* α-subunit. Amino acid residues using single letter designation, lower case residue less than 5/95 sequences. Number in total dominant column indicates the residue is >86 (90%) one amino acid in the 95 sequences. First amino acid in the list is the more common.

[3] Numbers at top of columns are the sum of occurrences for the column.
