# Supplementary material for: Multiple Amino Acid Sequence Alignment Nitrogenase Component 1: Insights into Phylogenetics and Structure-Function Relationships
Source: PLoS One. 2013 Sep 3;8(9):e72751. doi: 10.1371/journal.pone.0072751 (PMC3760896; doi:10.1371/journal.pone.0072751)
Supplement: Table S4 — Amino Acid Residue Variance in β-Subunit (Gene K). (PDF) [file pone.0072751.s005.pdf]

Table S-4. Amino Acid Residue Variance in β-Subunit (Gene K)[1,2,3]

Residue number	Invariant	Total Dominant >90%	Single variant, Dominant	Single variant	Double variant, Dominant	Double variant	Multiple variant, Dominant
	27	41	23	10	7	14	11
64				I/V			
65		91 N	N/d				
66		93 P	P/a				
70	C						
71		89 Q			Q/a/m		
72	P						
74	G						
75		92 A	A/s				
78						A/C/V	
81		94 G	G/a				
90				H/Q			
91	G						
92						S/G/a	
93		94 Q	Q/h				
94	G						
95	C						
98				Y/F			
100	R						
105				R/Q			
106		91 H					H (i,n,v)
108						K/R/q	
109				E/D			
117		94 S	S/a				
119						T/H/s	
120	E						
124	V						
125		89 F			F/Y/l		
126	G						
127				G/A			
130				N/R			
134						G/A/s	
142		90 Y	Y/f				

Table S-4, continued

Residue number	Invariant	Total Dominant >90%	Single variant, Dominant	Single variant	Double variant, Dominant	Double variant	Multiple variant, Dominant
151		88 T	T/S				
152		88 T	T/S				
153	C						
156	E						
157						V/T/I	
158		93 I			I/l/v		
159	G						
160		94 D	D/e				
161	D						
186		90 T			T/c/q		
187	P						
188				S/A			
189				F/Y			
191		89G					G (a,e,m,s)
192		91 S,T					S (h,n,t)
193						H/M/Q	
196	G						
197						Y/F/W	
229	G						
235						G/A/s	
236						D/N/s	
241		88 K					K (g,q,r)
256				D/E			
262		88 D					D (m,n,y)
264		91 P	P/g				
274		91 G					G (a,e,k,p)
276		92 T			T/l/a		
319	P						
321	G						
325		94 T	T/v				
326	D						
329						L/I/V	
340						I/V/p	
341		87 P					P (a,g,s,t)

Table S-4, continued

Residue number	Invariant	Total Dominant >90%	Single variant, Dominant	Single variant	Double variant, Dominant	Double variant	Multiple variant, Dominant
347		90 E					E (a,g,s,t)
348	R						
349		92 G	G/a				
351						L/A/v	
352						V/I/L	
353		94 D	D/n				
357		93 D	D/n				
365		88 K					K (l,q,r)
368		89 A			A/g/s		
371		94 G	G/a				
373		93 P	P/a				
374		94 D	D/s				
382		86 F					F (i,l,y)
390		93 P	P/v				
426	D						
440		94 D	D/e				
444		93 G	G/s				
448		88 G					G (a,c,l)
465		92 P			P/k/g		
468	R						
472		93 P	P/f				
475	D						
476	R						
486		94 G	G/f				
487	Y						
489	G						

[1] Based upon core sequence alignment, 95 sequences. Co-aligned residues: 62-142; 143-170; 177-180; 182-210; 225-233; 235-250; 252-257; 259-265; 270-273; 274-298; 300-306; 309; 311-316; 319-357; 358-359; 361-397; 398; 400-410; 420-422; 426-433; 435; 439-448; 449-455; 464-508 *A. vinelandii* , β-subunit numbering.

[2] Residue numbers based upon the *Azotobacter vinelandii* β-subunit. Amino acid residues using single letter designation, lower case residue less than 5/95 sequences. Number in total dominant column indicates the residue is >86 (90%) one amino acid in the 95 sequences. First amino acid in the list is the more common.

[3] Numbers at top of columns are the sum of occurrences for the column.
