# Supplementary material for: Multiple Amino Acid Sequence Alignment Nitrogenase Component 1: Insights into Phylogenetics and Structure-Function Relationships
Source: PLoS One. 2013 Sep 3;8(9):e72751. doi: 10.1371/journal.pone.0072751 (PMC3760896; doi:10.1371/journal.pone.0072751)
Supplement: Table S5 — Properties of Nif genes in Groups III and IV. (PDF) [file pone.0072751.s006.pdf]

Table S-5. Properties of *Nif* genes in Groups III and IV

Species	Group	Sec	N_2fix[1]	NifD[2] YP_001097187.1	NifK[2] YP_001097188.1	NifE[2] YP_001097189.1	NifN[2] YP_001097190.1	Cofactor Residue[3]		
				Bit Score[4]	Bit score[4]	Bit score[4]	Bit score[4]	360	380	441
Caldicellulosiruptor saccharolyticus DSM 8903	III-01		C	498	406	***349*** 272	***370***	V	T	S
Candidatus Desulforudis audaxviator MP104C	III-02	*	C	424	417	319	***353***	V	T	S
Desulfotomaculum kuznetsovii DSM 6115	III-03	*	P/C	399	430	323 ***294***	***379***	V	T	S
Methanocaldococcus sp. FS406-22	III-04		*C	399	395	348 ***282***	***333***	T	T	S
Methanococcus aeolicus Nankai-3	III-05		*	373	384	273 ***264***	***318*** 234	T	T	S
Methanococcus maripaludis C5	III-06		*	985	947	991	946	S	L	I
Methanothermobacter thermautotrophicus Delta H	III-07		*	692	606	529	437 ***363***	N	M	I
Thermodesulfatator indicus DSM 15286	III-08	*	C	402	379	***319*** 249	***304***	V	T	S
Oscillochloris trichoides DG6	IV-01			401	347	***325***	***353***	T	T	G
Roseiflexus sp. RS-1	IV-02		C	392	359	***328***	***357***	V	T	G
Roseiflexus castenholzii DSM 13941	IV-03		C	379	356	***315***	***352***	V	T	G

1. From Dos Santos, et al. [33] identification as C = Group C, P = potential nitrogen fixer, and * = demonstrated nitrogen fixer. *Desulfotomaculum kuznetsovii DSM 6115* was listed as a potential nitrogen fixer (P) although it is missing NifN which would make it "C" by Dos Santos, et al. criterion.
2. Search query protein using authentic protein for each category from III-06 *Methanococcus maripaludis C5.*
3. Amino acid residue at specified sequence position in the cofactor pocket, see Table 9.
4. Bit scores are for the proteins from the indicated species. ***Bold/italics*** bit scores are for the structural protein homologue, where NifE probe returns NifD structural protein and NifN returns NifK structural protein. For example, authentic NifE probe III-06 *Methanococcus maripaludis C5* (YP_001097189.1) returns NifD (bit = ***349***) and NifE (bit = 272) for species III-01 *Caldicellulosiruptor saccharolyticus DSM 8903*.
