# Supplementary material for: Multiple Amino Acid Sequence Alignment Nitrogenase Component 1: Insights into Phylogenetics and Structure-Function Relationships
Source: PLoS One. 2013 Sep 3;8(9):e72751. doi: 10.1371/journal.pone.0072751 (PMC3760896; doi:10.1371/journal.pone.0072751)
Supplement: Table S6 — Strong Motifs in Core Alignment α-subunit (Gene D). (PDF) [file pone.0072751.s007.pdf]

Table S-6. Strong Motifs in Core Alignment α-subunit (Gene D)*

Residue Number	Group I (9)	Group II (7)	Group III (2)	Group IV (31)	Group Mo (3)	Group Anf (23)	Group Vnf (15)
17				M		H	
32						A	
48						G	
49						Y	
50						L	
53				T			
65					A	(C)	(C)
69					G	H	L
74							V
95				Y			
97				P			
99						I	
115				V			
119				N			
144		P					
152				N		Q	
153	E						
163				G			
165				D			
193	L	A		M		G	
197						K	
198						I	
200	D	N		E			
203	R						
221				R			
225						Y	
232			D	K			
233				N			
235				I			
236				R		E	
238				F			
240				Y			
252							H
264				I			
269				A			
274						E	N
279		I					

Table S-6 (continued)

Residue Number	Group I	Group II	Group III	Group IV	Group Mo	Group Anf	Group Vnf
280							G
296	E			N			
299						G	
355				Q			T
358	L						
361	R	H					
363		Y					
364						A	T
376				T			
377							M
378						Y	
385						G	
387					Y		
389		G					
390						G	V
394						C	G
400							Y
406				V			
416				R			
422				I			
425				N			P
427						P	V
428							
432				Y			
435							H
443				T			
444	W						
445			E				
446				T			
451	H					K	M
458						R	
479				A			

* Residue numbers are for *A. vinelandii* α-subunit; (#) are number of strong motif residues in the Group; residue 65 in Anf and Vnf Groups is cysteine in both, not a strong motif.
