# Supplementary material for: Multiple Amino Acid Sequence Alignment Nitrogenase Component 1: Insights into Phylogenetics and Structure-Function Relationships
Source: PLoS One. 2013 Sep 3;8(9):e72751. doi: 10.1371/journal.pone.0072751 (PMC3760896; doi:10.1371/journal.pone.0072751)
Supplement: Table S7 — Strong Motifs in Core Alignment β-subunit (Gene K). (PDF) [file pone.0072751.s008.pdf]

Table S-7 Strong Motifs in Core Alignment β-subunit (Gene K)*

Residue number	Group I (3)	Group II (2)	Group III (1)	Group IV (30)	All Nif (2)	Group Anf (3)	Group Vnf (1)
71				A			
79				N			
80				Y			
87				T			
88		H					
89		S		N			
99				P			
101			Y	H			
105					R	(Q)	(Q)
118	M						
121				K			
123				T			
127						A	
139				W			
144					P		
145							R
148				M			
154	M						
169				F			
185				K			
200				F			
209				P			
250				H			
253				W			
256						E	
265				Y			
286				K			
294						N	
305				Y			
338				H			
349				A			
363				T			
365				L			
400				R			
410				T			
428				H			
431	R						
474				E			
506				A			
507				T			

*Residue numbers are for *A. vinelandii* β-subunit; (#) number of strong motif residues in the Group; residue 105 in Anf and Vnf Groups is conserved Q in both, not a strong motif.
