# Supplementary material for: Multiple Amino Acid Sequence Alignment Nitrogenase Component 1: Insights into Phylogenetics and Structure-Function Relationships
Source: PLoS One. 2013 Sep 3;8(9):e72751. doi: 10.1371/journal.pone.0072751 (PMC3760896; doi:10.1371/journal.pone.0072751)
Supplement: Table S8 — α, β-Subunit Residues within 5 Å any Atom in P-cluster. (PDF) [file pone.0072751.s009.pdf]

Table S-8. α, β-Subunit Residues within 5 Å any Atom in P-cluster*

	Group I		Group II		Group III		Group IV		Anf		Vnf		All	
Residue	Invar	Variant	Invar	Variant	Invar	Variant	Invar	Variant	Invar	Variant	Invar	Variant	Invar	Variant
α-62	C		C		C,U		C		C		C		C,U	
α-64		Y,F		Y,F		Y(f)	Y			Y(f)		F,Y		Y,F
α-85	P		P		P		P		P		P		P	
α-86		V,I		I,V		V(a)	I			V,I,(n)		V,L,I		V,I,L,(n)
α-87	G		G		G		G		G		G		G	
α-88	C		C		C		C		C		C		C	
α-91		Y,W,(l)		Y,(f)		Y,G	F		D			D,N		Y,W,(l),F,G,D,N
α-92	S			S,T,A		T,(n,y)	S		T		T			T,S,(n,y),A
α-152		S,(c)		A,S		A,T,(l)	N		Q		T			S,C,A,(l),T,N,Q
α-153	E		T			T,(n)	T		T		T			E,T,(n)
α-154	C		C		C		C		C		C		C	
α-155	P		P			T,P,(s)	S			A,(t)		P,A,T		P,(t,s),A
α-184	E		E		P		E		P			P,A		E, P, A
α-185	G		G		G		G		G		G		G	
α-186		F,(l)	Y			F,C,(y)	F		F		F			F,Y,C, L
β-70	C		C		C		C		C		C		C	
β-72	P		P		P		P		P		P		P	
β-92	S		S			S,A,(g)	S		G		G			S,A,G
β-93		Q,(h)	Q		Q		Q		Q		Q			Q(h)
β-94	G		G		G		G		G		G		G	
β-95	C		C		C		C		C		C		C	
β-98	Y			Y,(f)		Y,F	Y		F		F			F, Y
β-99		F,Y		H,L		V,(a)	P		V		V			F,Y,H,L,(a),P,V
β-151		T,(s)		T,S	T		S			T,(s)	T			T, S
β-152		T,S	T		T		T		T		T			T,S
β-153	C		C		C		C		C		C		C	
β-154	M			L,V,(s)	S		L			S,C	S			M,V,L,S,C
β-188		A,S	S			S,(a)	S		S		S			S,A

*Residue numbers are for *A. vinelandii* subunits. Lower case () indicates a single occurrence. The order of residues indicates relative number of occurrences. For U is Sec, see text for discussion.
