# Supplementary material for: Multiple Amino Acid Sequence Alignment Nitrogenase Component 1: Insights into Phylogenetics and Structure-Function Relationships
Source: PLoS One. 2013 Sep 3;8(9):e72751. doi: 10.1371/journal.pone.0072751 (PMC3760896; doi:10.1371/journal.pone.0072751)
Supplement: Table S9 — Residues in α-Subunit within 5 Å of Any Atom of Metal Cluster Component of FeMoco. (PDF) [file pone.0072751.s010.pdf]

Table S-9. Residues in α-Subunit within 5 Å of Any Atom of Metal Cluster Component of FeMoco*

	Group I		Group II		Group III		Group IV		Anf		Vnf		All	
**Residue**	**Invar**	**Variant**	**Invar**	**Variant**	**Invar**	**Variant**	**Invar**	**Variant**	**Invar**	**Variant**	**Invar**	**Variant**	**Invar**	**Variant**
70	V		V		V		V		V		V		V	
96	R		R			R,K	R		K		K			R,K
191	Q		Q		Q		Q		Q		Q		Q	
195	H		H		H		H		H		H		H	
229		Y,(h)	Y		Y		Y		Y			Y,(f)		Y,(f,h)
231	I		I			I,M	I		I		I			I,M
275	C		C		C		C		C		C		C	
277	R		R		R		R		R		R		R	
278	S		S		S		S		S		S		S	
355		V,I, (m,l)		V,A		V,(a,s)	Q			P,(s)	T			V,I,P,T,S,(a,l,m)
356	G		G		G		G		G		G		G	
357	G		G			G,a	G		G		G			G,a
358	L		S		P		P		S		P			L,S,P
359	R		R			R,K	R		K		R			R,K
360		P, S		A,S,(g)		V,T,(s,n)		V,(t)	L		L			P,L,S,AV,T,(n,g)
380	E			E,Q		T,L,M	T		K		K			E,K,T,Q,(l,m)
381	F		F		F		F		F		F		F	
441	M			M,L,I		S,I	G			A (i,v)	G			M,L,G,A,I,S,(v)
442	H		H		H		H		H		H		H	
residues	16		15		10		18		17		18		9	

*Residue numbers are for *A. vinelandii* α-subunit. Lower case () indicates a single occurrence. The order of residues indicates relative number of occurrences.
