# Supplementary material for: Multiple Amino Acid Sequence Alignment Nitrogenase Component 1: Insights into Phylogenetics and Structure-Function Relationships
Source: PLoS One. 2013 Sep 3;8(9):e72751. doi: 10.1371/journal.pone.0072751 (PMC3760896; doi:10.1371/journal.pone.0072751)
Supplement: Table S10 — Residues Within 5 Å of Any Atom of Homocitric Acid Component of FeMoco. (PDF) [file pone.0072751.s011.pdf]

Table 10. Residues Within 5 Å of Any Atom of Homocitric Acid Component of FeMoco*

	Group I		Group II		Group III		Group IV		Anf		Vnf		All	
Residue	Invar	Variant	Invar	Variant	Invar	Variant	Invar	Variant	Invar	Variant	Invar	Variant	Invar	Variant
α-65	A		A		A		A		C		C			A,C
α-95x		G,Q,S,A,N,T		T,(g)		T,S	Y		T					G,T,Q,S,A,N,Y
α-96	R		R			R,K	R		K		K			R,K
α-191	Q		Q		Q		Q		Q		Q		Q	
α-380x	E			E,Q		T,(l,m)	T		K		K			E,K,T,Q,(l,m)
α-424	G		G		G		G		G		G		G	
α-425		I,V		I,V		L,I	N			K,V	P			I,V,L,NK,P
α-426x	K			K,R	K		K		R		R			K,R
α-427x	E			D,E	E		E		P		V			E,D,V,P
α-440x	Q		Q			N(l,m)	N		N		N			Q,N,(l,m)
α-442	H		H		H		H		H		H		H	
β-98x	Y			Y,(f)		Y,F	Y		F		F			Y,F
β-101x		S,T,(a,n)	M,S,(t)		Y		H			M,L	L			S,T,M,L,H,(a,n)
β-105x	R		R		R		R		Q		Q			R,Q
Residues	11		7		8		14		12		13		3	

*Residue numbers are for *A. vinelandii* subunit. Lower case () indicates a single occurrence. The order of residues indicates relative number of occurrences. x indicates residues contact homocitric acid only through water H-bonds.
